# Effectiveness of Family Meetings for Family Caregivers on Delaying Time to Nursing Home Placement of Dementia Patients: A Randomized Trial

**DOI:** 10.1371/journal.pone.0042145

**Published:** 2012-08-02

**Authors:** Karlijn J. Joling, Harm W. J. van Marwijk, Henriëtte E. van der Horst, Philip Scheltens, Peter M. van de Ven, Bregje A. Appels, Hein P. J. van Hout

**Affiliations:** 1 Department of General Practice and the EMGO Institute for Health and Care Research, VU University Medical Center, Amsterdam, The Netherlands; 2 Department of Neurology, Alzheimer Center, VU University Medical Center, Amsterdam, The Netherlands; 3 Department of Epidemiology and Biostatistics, EMGO Institute for Health and Health Care Research, VU University Medical Center, Amsterdam, The Netherlands; 4 Medical Psychology, Slotervaart Hospital, Amsterdam, The Netherlands; Federal University of Rio de Janeiro, Brazil

## Abstract

**Background:**

Interventions relieving the burden of caregiving may postpone or prevent patient institutionalization. The objective of this study was to determine whether a family meetings intervention was superior to usual care in postponing nursing home placement of patients with dementia.

**Methods:**

A randomized multicenter trial was conducted among 192 patients with a clinical diagnosis of dementia living at home at enrolment and their primary family caregiver. Dyads of caregivers and patients were randomized to the family meetings intervention (n = 96) or usual care (n = 96) condition. The intervention consisted of two individual sessions with the primary caregiver and four family counseling sessions that included family members and friends. The primary outcome measure was the time until institutionalization of the patient. Intention-to-treat as well as per protocol analyses were performed. Survival analyses were carried out to evaluate the effectiveness of the intervention.

**Results:**

During 18 months follow-up 23 of 96 relatives with dementia of caregivers in the intervention group and 18 of 96 relatives with dementia of caregivers in the usual care group were institutionalized. No significant difference between the intervention and the usual care group was found in time until institutionalization (adjusted hazard ratio (HR) 1.46, 95% confidence interval (CI) 0.78 to 2.74). The per-protocol analysis revealed no significant effect either (adjusted HR 0.57, 95% CI 0.21 to 1.57), although the number of placements among the adherers was relatively low (9.4%). A subgroup effect was found for patients’ age, with a significantly higher risk of institutionalization for ‘younger’ patients in the intervention group compared with the usual care group (adjusted HR = 4.94, 95% CI 1.10 to 22.13).

**Conclusion:**

This family meetings intervention for primary caregivers of patients with dementia did not postpone patient institutionalization more than usual care.

**Trial Registration: **Controlled-Trials.com ISRCTN90163486**:**

## Introduction

With the number of persons with dementia estimated to double every twenty years, the burden of dementia care will be difficult to bear for families and health care systems worldwide [1], [2]. Most patients with dementia still live at home and are cared for by a family member [3], [4]. Caring for these patients at home, however, is associated with a high burden and an increased risk of mental health problems. Approximately 20% of patients is institutionalized in the first year after dementia has been diagnosed. This rises to 50% after five years and approaches 90% after eight years [5].

A poor health of the caregiver is one of the most important predictors for nursing home placement of demented persons [5]–[8]. Since institutionalization of patients is a heavy cost driver, any intervention that can relief the burden of caregiving and (thereby) delay nursing home placement is likely to be very cost-effective. Several caregiver interventions targeting psychosocial factors have proven to be effective in helping caregivers to postpone nursing home placement of patients with mild to moderately severe dementia when compared with usual care [9], [10]. The essential components of effective interventions were individual targeting, psycho-education, counseling and support [11]–[13]. A systematic review reported a risk reduction of 0.33 after 6 or 12 months of intervention, which indicates 33% less institutionalization compared to the minimal support or usual-care control group [10]. Furthermore, one RCT which tested the effectiveness of a counseling intervention including family meetings, found that in the first year after intake the intervention group had less than half as many nursing home placements as the control group [13]. After a period of 11 years, the intervention led to a substantial median delay in nursing home placement of 557 days (or approximately 1.5 years) compared to the control group while maintaining a comparable quality of life for the caregivers [14]. This intervention was developed to mobilize support of naturally existing family networks and consisted of individual counseling sessions, family meetings, support group participation and continuous availability of ad hoc telephone counseling.

In the Netherlands, several supportive services for family caregivers are available, but family meetings are rarely organized and never in a structured way. It is not clear yet how much value family meetings may have on top of usual care when implemented in real life practice. To estimate this, we conducted a randomized trial among primary family caregivers of community dwelling demented patients investigating the effectiveness of structured family meetings in comparison with usual care on postponing patient institutionalization. The effects of the intervention on preventing the development of depression and anxiety in caregivers are reported elsewhere [15].

## Methods

The protocol for this trial and supporting CONSORT checklist are available as supporting information; see Checklist S1 and Protocol S1. The design of this study has been described in detail elsewhere [16].

### Participants

Patients and their primary caregivers were recruited through memory clinics (n = 91), services delivering case management (n = 79), general practices, home care settings and meeting centers for people with dementia and their caregivers (n = 22) across the Netherlands. Caregivers were eligible if they were the primary family caregiver of a community dwelling relative with a clinical diagnosis of dementia and had at least one other family member or friend available to participate in the family meetings. If there was more than one family caregiver caring for the patient, the primary caregiver was identified as the person who coordinated the caring process, usually the person who spends the most hours on caregiving tasks. Caregivers were excluded when 1) they met the criteria for a clinical depressive or anxiety disorder as measured with the Mini International Neuropsychiatric Interview (MINI) [17], 2) the patient was already scheduled to move to a nursing home in short notice, 3) they had severe somatic or psychiatric co-morbidity which would impair participation in the study, or 4) they had insufficient language proficiency in Dutch for adequate participation in the family meetings and interviews. Written informed consent was obtained from all participants. In case of mental incompetence of a patient the family caregiver signed the consent for the patient.

### Ethics

The Medical Ethics Committee of the VU University Medical Centre approved the study protocol.

### Intervention

Caregivers randomized to the intervention group were invited to participate in six in-person counseling sessions: one individual preparation session, followed by four structured meetings that included their relatives and/or friends, and one additional individual evaluation session. The family meetings were held once every 2 to 3 months in the year following enrollment in the program.

### Preparation Meeting

The first individual session was aimed to prepare the caregiver for the family meetings and to propose the idea of seeking help from family and friends.

#### Family meetings

The aim was to offer psycho-education, teach problem solving techniques and mobilize the existing family networks of the patient and primary caregiver in order to improve emotional and instrumental support. The content of the sessions was guided by the needs of the caregiver. During the first family meeting the purpose of the meetings, the protocol, ground rules and the counselor’s role were explained to the caregiver and the family. Relevant issues were identified (e.g. management of patient behavioral problems, coping with feelings of guilt) and the counselor motivated the family to form ideas to help the caregiver and to delegate tasks. The follow up meetings reviewed the previous session, previous commitments and the progress of tasks. Ad hoc telephone counseling from the same counselor was available continuously to caregivers and their families beyond the scheduled sessions and was provided on demand of the caregiver.

#### Evaluation session

After the final family session, an individual session was held to evaluate the caregiver’s satisfaction with the intervention program and to start additional support when requested.

The counselors who led the family meetings had an advanced degree in nursing, social work, psychology or an allied profession and were trained prior to the study by the research team. One counselor was assigned to each caregiver to establish an ongoing relationship with a person familiar with the situation. The family meetings were audio taped for supervision and reviewed randomly and on request to give feedback to the counselors. To encourage and evaluate protocol adherence, after a family session, the counselor filled in a standardized form and was contacted by the researcher (KJJ) individually to monitor and discuss difficulties. More detail about the intervention can be found elsewhere [15], [16].

#### Usual care

Caregivers randomized to the control condition received care as usual. Usual care in the Netherlands may consist of a range of health care and welfare services and can differ across participants. However, family meetings are rarely organized and never in a structured way or with follow-up sessions. They also tend to focus on providing clinical information and not on increasing family support and relieving the caregiver. Usual care participants were free to use all types of care, including community-based mental health services or support resources other than family meetings at any time throughout the 18 months follow-up, therefore reflecting standard care.

### Primary Outcome

The primary outcome for this analysis was the time to nursing home placement of the patients in calendar days. We obtained information on institutionalization of the patient from the primary caregiver over 18 months of follow-up. Socio-demographics and clinical characteristics were collected for both patients and caregivers (Table 1). The participants’ use of health care services and their participation in family meetings were recorded.

**Table 1:** Baseline demographic and clinical characteristics of the caregivers and the patients.

	Intervention (n = 96)	Usual Care (n = 96)
**Patient**		
Age, mean (SD)	72.8 (9.1)	76.7 (8.3)
Female gender, n (%)	30 (31.3)	32 (33.3)
Educational level, n (%)		
Elementary/Lower	42 (43.8)	44 (45.8)
Secondary/Higher/University	54 (56.3)	50 (52.1)
ADL independencies (out of 6), mean (SD)	5.1 (1.4)	5.3 (1.1)
IADL independencies (out of 7), mean (SD)	2.7 (1.8)	2.6 (1.5)
MMSE (0–30), mean (SD)	21.4 (4.9)	21.7 (5.6)
Neuropsychiatric symptom severity (NPI-Q), mean (SD)	8.5 (5.4)	9.5 (6.3)
Years since clinical diagnosis, mean (SD)	1.25 (1.25)	1.02 (1.06)
Type of dementia, n (%)		
Alzheimer disease	54 (56.3)	56 (58.3)
Vascular dementia/Mixed	24 (25.0)	21 (21.9)
Frontotemporal dementia	5 (5.2)	6 (6.3)
Lewy body/Parkinson dementia	9 (9.4)	8 (8.3)
Type not specified/unknown	4 (4.2)	5 (5.2)
**Caregiver**		
Age, mean (SD)	67.8 (9.8)	71.2 (10.7)
Female gender, n (%)	67 (69.8)	68 (70.8)
Spouse of the patient, n (%)	92 (95.8)	89 (92.7)
Living with patient, n (%)	93 (96.9)	91 (94.8)
Educational level, n (%)		
Elementary/Lower	28 (29.2)	34 (35.4)
Secondary/Higher/University	66 (68.7)	62 (64.6)
Caregiver distress (NPI-Q distress score), mean (SD)	11.6 (8.1)	12.6 (9.1)
Depressive symptoms (CES-D score), mean (SD)	12.1 (7.9)	10.8 (7.1)
Anxious symptoms (HADS-A score), mean (SD)	6.1 (3.4)	4.8 (3.5)

Abbreviations: SD: Standard deviation; (I)ADL: (Instrumental) activities of daily living; MMSE: Mini Mental State Examination; NPI-Q: Neuropsychiatric Inventory- Questionnaire; CES-D: Centre for Epidemiologic Studies Depression Scale; HADS-A: Hospital Anxiety and Depression Scales.

### Power Calculation

The study was primarily designed and powered to derive a relevant effect on the outcomes incidence of depression and anxiety in caregivers [15]. We recalculated the power for the primary outcome of this paper, time until institutionalization. In our cohort of 96 patient-caregiver dyads per group, with 18 months of follow-up time, a two-sided test with α = 0.05, we derived a power of 76% to demonstrate a comparable effect on institutionalization as in the previous study of Mittelman et al. In that study 11% of the patients in the intervention group was institutionalized compared to 23% of the patients in the control group after 12 months of follow up [13]. In our calculation we assumed exponential distribution for the time to institutionalization in both arms.

### Randomization & Blinding

After informed consent and baseline measurements, to secure allocation concealment, an independent researcher who was not involved in the trial conducted randomization according to a computer generated allocation sequence. Patients and their primary family caregiver were randomized as a dyad, stratified by recruitment centre, in blocks of four to either usual care or the family meetings intervention. The interviewers who measured the outcomes were blinded to group allocation. The participants and the counselors conducting the family meetings were aware of the intervention assigned.

### Statistical Analysis

We compared the baseline characteristics of dropouts and those who completed the 18-month measurement by performing logistic regression analysis. Survival analyses using the Kaplan-Meier method and Cox proportional hazard regression were carried out to evaluate the effectiveness of family meetings compared to usual care on the primary outcome measure. The unadjusted difference in time until institutionalization between both groups was tested with the log rank test. Adjusted differences were expressed as hazard ratios (HRs) and corresponding 95% confidence intervals (CI) for the intervention group, compared to the usual care group. Time to event was measured in days from date of the baseline assessment to the date of admission to a long-term care institution. For patients who died without prior institutionalization, date of death was used as a censoring event. Patients who were still living in the community at the end of the study period were censored at the date of the 18 months follow-up assessment. For patients who were lost to follow up, the date of the last contact was used as the censoring date. We conducted a sensitivity analysis to examine the effect of censoring patients who died without ever being placed. All analyses were conducted according to the intention-to-treat principle. The age of the patient was incorporated as covariate in the analysis, because this variable differed significantly between the intervention and usual care group at baseline and was also significantly associated with the primary outcome.

We also performed a per protocol analysis, comparing the outcomes of “adherers” to the intervention protocol (those who participated in at least three family meetings within 18 months) with the usual care group. Finally, we conducted effect modification analyses to determine whether the intervention resulted in different effects for relevant subgroups of patients and their caregivers. We investigated the possible interaction effects of the intervention with the patient characteristics age, gender, level of cognitive function (assessed with the Mini Mental State Examination), type of dementia (Alzheimer’s disease versus other types of dementia), with caregiver distress (assessed with the Neuropsychiatric Inventory Questionnaire distress score), and with recruitment from sites offering intensive support resources. Continuous variables were dichotomized at the median in these analyses. All analyses were performed with the SPSS (version 15.0) and STATA (version 11) statistical packages. Statistical significance was considered as two-tailed p<0.05.

## Results

### Participant Flow and Recruitment

Participants were recruited from November 2007 to November 2009. Of the caregivers assessed for eligibility, 192 met all inclusion criteria and were willing to participate. Reasons for exclusion were ‘patient not diagnosed with dementia’ (n = 10), ‘no other family member or friend available to participate in the family meetings’ (n = 16), ‘insufficient command of the Dutch language’ (n = 9), ‘patient was (scheduled to be) institutionalized’ (n = 39), ‘caregiver had a clinical depressive or anxiety disorder at intake’ (n = 7).

**Figure 1:**
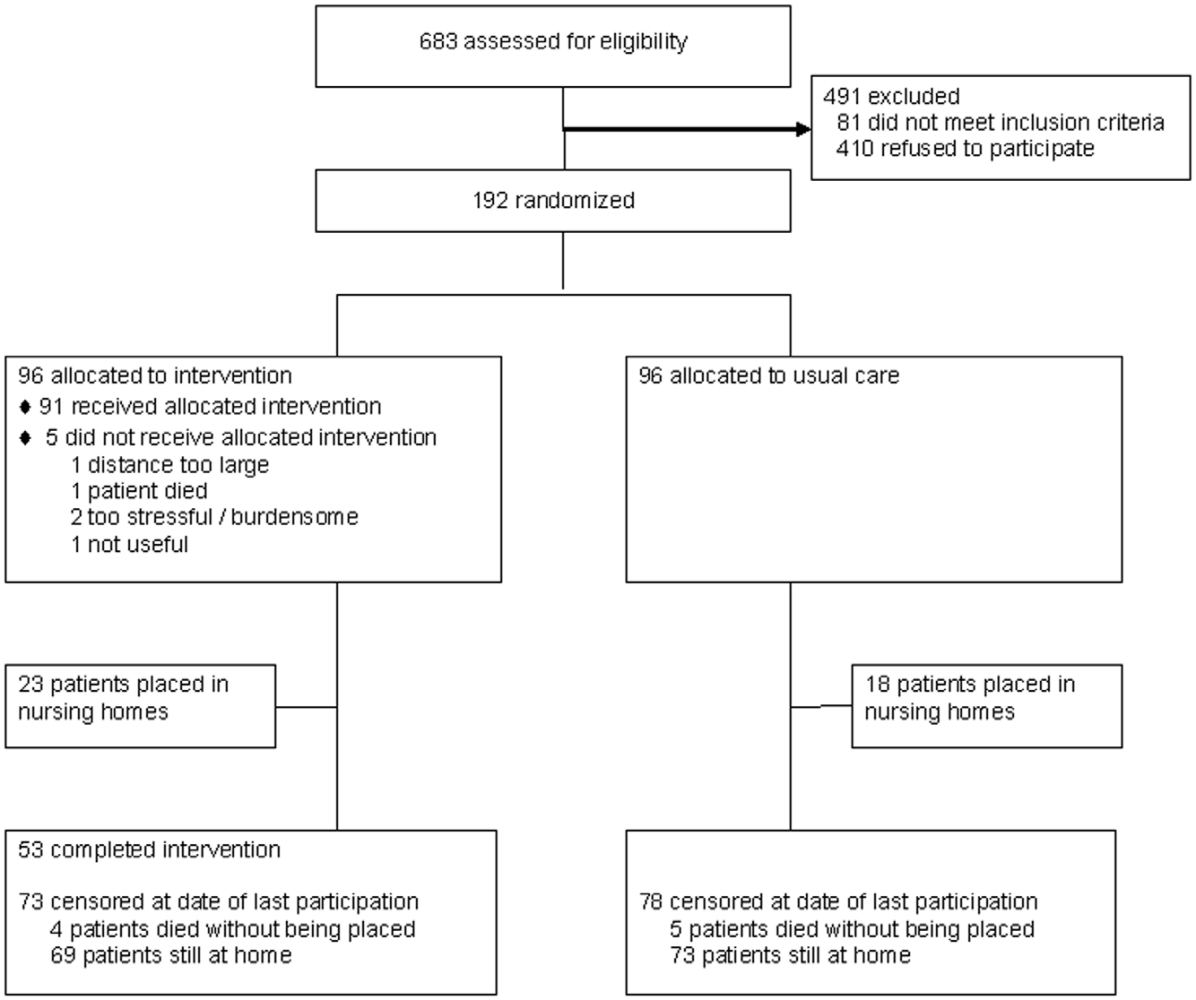
Flow diagram of the study sample. Information about the status of the patient (placed, deceased, still at home) and date of placement was known for all patients.

A substantial number of 410 caregivers refused to participate. Most refusals were due to claiming a lack of need for this intervention (n = 202). These caregivers already used other services, said they could (still) manage on their own or did not expect to benefit from the intervention. Other reasons for refusal included: too burdensome (n = 85), practical reasons (n = 33), resistance of the family or patient (n = 21), not willing to burden their family (n = 19), difficulties with the randomized design (n = 3) and reason unknown (n = 47). Participants were equally randomized to the intervention (n = 96) or usual care group (n = 96) (Figure 1).

### Baseline Characteristics

Table 1 presents the socio-demographic and clinical characteristics of the caregivers and patients at baseline. Imbalances were found between the intervention and usual care group on three of the baseline variables. Patients and caregivers in the intervention group were significantly younger (patient’s age: difference in mean = 3.85, 95% CI 1.37 to 6.33, p = 0.002 and caregivers’ age: difference in mean = 3.37, 95% CI 0.45 to 6.30, p = 0.024) and caregivers had higher levels of anxious symptoms (HADS-A score) (difference in mean = −1.24, 95% CI −.22 to −0.27, p = 0.013) at baseline than participants in the usual care group.

### Numbers Analyzed

For the primary outcome –time until institutionalization- follow-up data on all of the patients were available and hence all participants were included in the intention-to-treat analyses.

### Outcomes and Estimation

**Figure 2:**
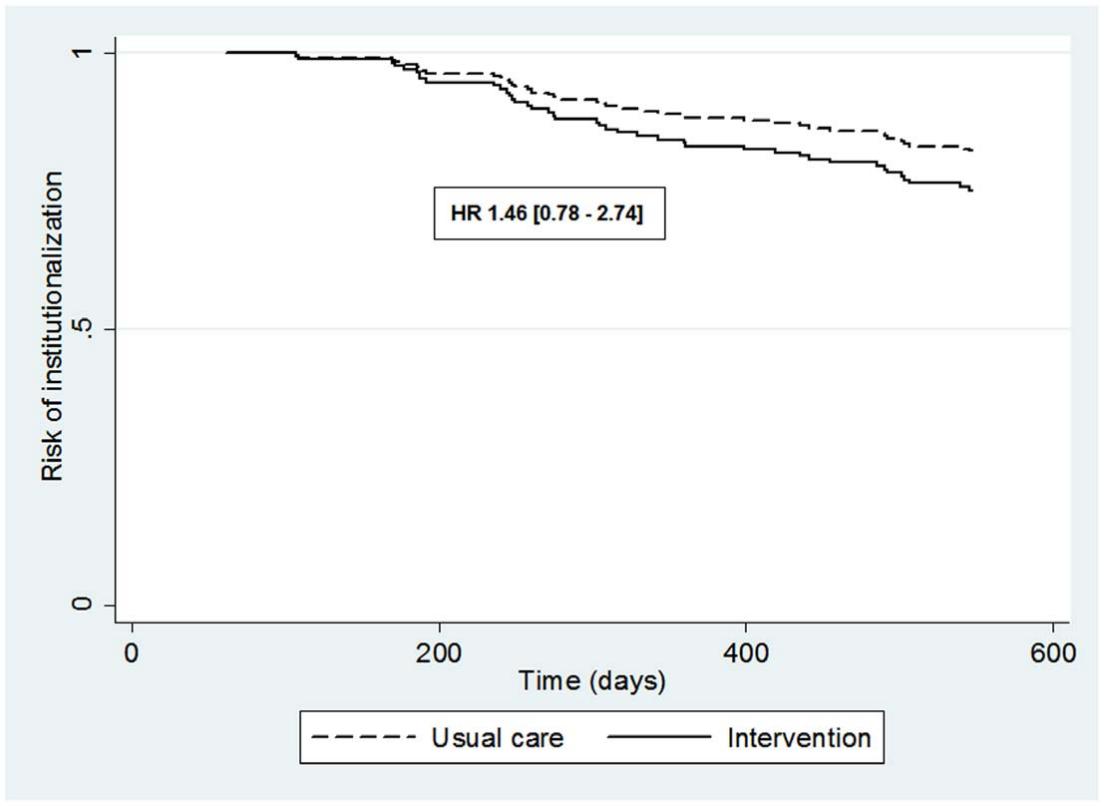
Survival curve of time until nursing home placement of patients in the 18 months of follow-up in the usual care and intervention group as estimated from the adjusted Cox proportional hazard model.

There were 41 (21.4%) nursing home placements of patients within 18 months of enrollment into the study: 23 in the intervention group and 18 in the usual care group (χ^2^ = 0.78, 1 df, p = 0.38). Analysis showed no superior overall effect of the family meetings intervention on days until patient institutionalization (χ^2^ log rank = 0.58, 1 df, p = 0.45). The unadjusted Hazard Ratio was 1.27 with a 95% CI ranging from 0.69 to 2.35 and p = 0.448. Table 2 presents the outcomes of the model adjusted for patients’ age, because this variable differed significantly between the intervention and usual care group at baseline and was also significantly associated with the primary outcome. The survival curves of the intervention and usual care group are displayed in Figure 2.

**Table 2:** Effects of the family meetings intervention on patient institutionalization.

	Number ofplacements (%)	HazardRatio*	95% CI	Riskdifference*	95% CI
*ITT analysis*					
Intervention (n = 96)	23 (24.0)	1.46	0.78–2.74	0.08	−0.04–0.20
Usual Care (n = 96)	18 (18.8)				
*PP analysis*					
Intervention adherers (n = 53)	5 (9.4)	0.57	0.21–1.57	−0.06	−0.18–0.07
Usual Care (n = 96)	18 (18.8)				

Results from the intention-to-treat and per protocol analyses.

Abbreviations: ITT: Intention to treat, PP: Per protocol, CI: Confidence interval.

* Intervention group versus usual care group, adjusted for age of the patient.

### Intervention Uptake and Per Protocol Analyses

Of the caregivers randomized to the intervention group, 95% (91/96) participated in the preparation session, and 76% (73/96) attended the family meetings. The majority of the intervention program (i.e. preparation session plus at least 3 family meetings) was completed by 55% (53/96) of the caregivers. Reasons for non-adherence were: resistance of family members/family conflicts (n = 11), too burdensome (n = 9), no perceived need for (more) family meetings (n = 8), placement in nursing home or death of the patient (n = 7), practical considerations (n = 5), other reasons (n = 3). The adherers did not significantly differ on most baseline characteristics compared with the nonadherers, only the patient’s number of ADL dependencies was slightly higher for non-adherers (OR = 1.47, 95% CI: 1.047 to 2.072, Wald χ^2^ = 4.95, df = 1, *p* = 0.026).

The per protocol analysis compared the adherers (n = 53) with the usual care group (n = 96), resulting in a unadjusted HR of 0.45 (95% CI 0.17 to 1.20, p = 0.111). Although the number of placements among the adherers was relatively low (9.4%), the analysis showed no significant difference compared with the usual care group either. The outcomes of the adjusted analysis are presented in Table 2.

Of the 73 caregivers who attended at least one family meeting, 64 completed an evaluation form after their last session. Satisfaction among the participating caregivers was high: 53 (83%) experienced the family meetings as useful, while 8 caregivers experienced no benefits (data on 3 persons were missing/inconclusive).

### Use of Health Care and Supportive Services

For 92 of the 96 caregivers in the usual care group and 89 of the 96 intervention caregivers data on the health care use and supportive services were available. We found that 52 in the usual care group received additional counseling from a psychologist, casemanager or social worker and 51 caregivers in the intervention group received such counseling (χ^2^ = 0.011, df = 1, p = 0.915). Twenty caregivers in the usual care group reported participation in a support group versus 19 caregivers in the intervention group (χ^2^ = 0.004, df = 1, p = 0.949).

### Ancillary Analyses

Four patients in the intervention group and five in the usual care group died without being placed in a nursing home and were censored at the day of death. We conducted two sensitivity analyses to examine the effect of censoring these patients. Both analyses showed no significant differences between the intervention and usual care group, so the conclusion drawn from the original analysis remains unchanged. In the first analysis, the nine patients were assumed to be institutionalized at the day of death (adjusted HR 1.34, z = 1.02, p = 0.307, 95% CI 0.760 to 2.369). A second sensitivity analysis was carried out in which the patients were assumed to live at home until the end of the study (adjusted HR = 1.48, z = 1.21, p = 0.225, 95% CI 0.786 to 2.774).

#### Effect modification analyses

To help guide clinical management, we investigated with effect modification analyses, whether subgroups with specific clinical (severity and type of dementia) characteristics would benefit (more) from family meetings and whether recruitment of patient-caregiver dyads via sites offering intensive support resources (casemanagement yes/no) modified the intervention effect. These analyses were pre-specified in the trial protocol [16]. Additionally, we performed three post-hoc analyses to explore possible effect modification of patient demographics (age and gender) and caregiver distress. For all subgroup analyses, we looked for interaction effects in the Cox regression analysis. We only found a significant interaction with patients’ age dichotomized at the median of 75.6 years (randomization*age interaction: adjusted HR = 0.16, 95% CI = 0.03 to 0.88, p = 0.034). For ‘younger’ patients (age<75.6 years) the risk of institutionalization was almost five times higher in the intervention group compared with the usual care group (adjusted HR = 4.94, 95% CI 1.10 to 22.13). For older patients (age ≥75.6 years) the intervention group had a slightly lower risk than the usual care group (adjusted HR = 0.87, 95% CI 0.39 to 1.94).

## Discussion

This study indicates that this type of family meetings for caregivers of dementia patients did not delay patient institutionalization within 18 months follow up compared to usual care. Previously, a NYU caregiver intervention showed that a counseling program including family meetings was effective in preventing nursing home placement. Analyses of data collected over an 18-year period indicated a median delay in placement of 1.5 years [14]. Our results are not in line with these positive findings.

There may be several explanations for the lack of effects in our study. First, standard care in the Netherlands provided to caregivers and patients is already intensive. Most recruitment sites provided intensive routine care such as case management and support groups, and other health care services were also accessible for caregivers. An explanation might be that the family meetings in addition to the already intensive standard care do not result in significant benefits.

It might also be argued that the intervention lacked sufficient intensity to influence this outcome. Family meetings might be more beneficial if delivered more intensively over a shorter period of time or in combination with other intervention components. However, since there are already many supportive services available to caregivers in the Netherlands, the contrast with usual care would still be small.

The lack of effects in our study may also be due to the fact that the participants’ adherence to the intervention was not optimal. We applied several strategies to maintain a high level of participation. The sessions were scheduled at the convenience of the family as much as possible and the counselors provided the family meetings in the caregivers’ homes if they were unable to leave the patient. Nevertheless, only about half of the intervention caregivers completed the majority of the sessions. This could also mean that this type of intervention is not what these caregivers perceive to need.

According to the audiotapes that were listened, the standardized forms that were completed by the counselors after every session and the contact between the research team and counselors during the intervention period, the counselors carried out the intervention as instructed. Furthermore, all counselors were uniformly trained, used a structured manual and may be assumed to be adequately qualified to lead the family meetings. Therefore, we assume that the actual quality of the intervention was no reason for the lack of effects in our study.

The results of our trial make a valuable contribution to the existing literature. Our study is the first study outside the original NYU development site to investigate the effectiveness of structured family meetings compared to usual care. Other strengths are the relatively large sample size and the lack of missing data on the primary outcome. For all patients, we were able to trace their status, also after caregivers stopped actively participating in the study. Considering the randomized design, the independent assessments, the concealed allocation and the adjustment for baseline differences associated with the outcome, it is unlikely that selection bias has influenced the results.

A subgroup analysis suggested that the ‘risk’ of institutionalization in ‘younger’ patients was higher among intervention participants than for participants receiving care as usual. This finding was unexpected. It may reflect that family counseling may boost the caring capacity of the caregiver and family, but it can also have precisely the opposite effect, particularly in younger caregivers, by making them realize at an earlier stage that their limits have been reached and that admission to a nursing home is appropriate. As we performed a post-hoc subgroup analysis this finding should be interpreted with caution.

Our study also has some limitations. Adherence to the intervention protocol was not optimal. Perhaps, a more multifaceted approach would have given better results. On the other hand, the results reflect the effectiveness in usual routine care for caregivers who were interested in this particular intervention. In view of the generalizability, it is also important to note that the mean age of our sample was rather low for a sample of patients with a diagnosis of dementia.

Another limitation involves the power of the study. The study was originally powered to derive a relevant effect on the development of mental disorders in the caregiver [16]. The somewhat limited power of 76% might explain why we were not able to detect a difference between the intervention and control group with regard to institutionalization. One possibility to increase power is to extend follow-up, which could provide a sufficient number of events (and time) to derive a significant effect.

In conclusion, although most caregivers participating in the family meetings intervention felt supported, the intervention was not more effective than usual care in delaying time until institutionalization of patients. An remaining question in psychosocial intervention research for caregivers of persons with dementia is what works for whom and what timing is appropriate? It might be that family meetings are more effective when targeted at those caregivers most in need. Based on our findings we cannot state which specific subgroups of caregivers might benefit most from this intervention. However, we did find that caregivers who were satisfied about the family meetings experienced a higher lack of family support at baseline than the other intervention caregivers [15]. This might indicate that family meetings should be targeted at the more ‘dysfunctional’ families, nonetheless delivering a more intensive program in which motivating caregivers should be an important component.

## Supporting Information

Checklist S1 CONSORT Checklist.
(PDF)

Protocol S1 Trial Protocol.
(PDF)

## References

[1] Ferri CP, Prince M, Brayne C, Brodaty H, Fratiglioni L, et al (2005) Global prevalence of dementia: a Delphi consensus study. Lancet 366: 2112–2117. 16360788 10.1016/S0140-6736(05)67889-0 PMC2850264

[2] Wimo A, Winblad B, Jonsson L (2010) The worldwide societal costs of dementia: Estimates for 2009. Alzheimers Dement 6: 98–103. 20298969 10.1016/j.jalz.2010.01.010

[3] AARP and National Alliance for Caregiving (1997) Family Caregiving in the US: Findings from a national survey. Bethesda, MD: AARP and National Alliance for Caregiving.

[4] Dutch Institute for Healthcare Improvement (2005) Guideline diagnostics and medicinal treatment of dementia. Utrecht: CBO/MWR. 75 p.

[5] Luppa M, Luck T, Brahler E, Konig HH, Riedel-Heller SG (2008) Prediction of institutionalisation in dementia. A systematic review. Dement Geriatr Cogn Disord 26: 65–78. 18617737 10.1159/000144027

[6] Gallagher D, Ni MA, Crosby L, Ryan D, Lacey L, et al (2011) Determinants of the desire to institutionalize in Alzheimer’s caregivers. Am J Alzheimers Dis Other Demen 26: 205–211. 21378063 10.1177/1533317511400307 PMC10845488

[7] Hebert R, Dubois MF, Wolfson C, Chambers L, Cohen C (2001) Factors associated with long-term institutionalization of older people with dementia: data from the Canadian Study of Health and Aging. J Gerontol A Biol Sci Med Sci 56: M693–M699. 11682577 10.1093/gerona/56.11.m693

[8] Spitznagel MB, Tremont G, Davis JD, Foster SM (2006) Psychosocial predictors of dementia caregiver desire to institutionalize: caregiver, care recipient, and family relationship factors. J Geriatr Psychiatry Neurol 19: 16–20. 16449755 10.1177/0891988705284713 PMC1361276

[9] Brodaty H, Green A, Koschera A (2003) Meta-analysis of psychosocial interventions for caregivers of people with dementia. J Am Geriatr Soc 51: 657–664. 12752841 10.1034/j.1600-0579.2003.00210.x

[10] Olazaran J, Reisberg B, Clare L, Cruz I, Pena-Casanova J, et al (2010) Nonpharmacological therapies in Alzheimer’s disease: a systematic review of efficacy. Dement Geriatr Cogn Disord 30: 161–178. 20838046 10.1159/000316119

[11] Belle SH, Burgio L, Burns R, Coon D, Czaja SJ, et al (2006) Enhancing the quality of life of dementia caregivers from different ethnic or racial groups: a randomized, controlled trial. Ann Intern Med 145: 727–738. 17116917 10.7326/0003-4819-145-10-200611210-00005 PMC2585490

[12] Lawton MP, Brody EM, Saperstein AR (1989) A controlled study of respite service for caregivers of Alzheimer’s patients. Gerontologist 29: 8–16. 2753374 10.1093/geront/29.1.8

[13] Mittelman MS, Ferris SH, Steinberg G, Shulman E, Mackell JA, et al (1993) An intervention that delays institutionalization of Alzheimer’s disease patients: treatment of spouse-caregivers. Gerontologist 33: 730–740. 8314099 10.1093/geront/33.6.730

[14] Mittelman MS, Haley WE, Clay OJ, Roth DL (2006) Improving caregiver well-being delays nursing home placement of patients with Alzheimer disease. Neurology 67: 1592–1599. 17101889 10.1212/01.wnl.0000242727.81172.91

[15] Joling KJ, van Marwijk HW, Smit F, van der Horst HE, Scheltens P, et al (2012) Does a family meetings intervention prevent depression and anxiety in family caregivers of dementia patients? A randomized trial. PLoS One 7: e30936. 22303473 10.1371/journal.pone.0030936 PMC3267736

[16] Joling KJ, Van Hout HP, Scheltens P, Vernooij-Dassen M, van den Berg B, et al (2008) (Cost)-effectiveness of family meetings on indicated prevention of anxiety and depressive symptoms and disorders of primary family caregivers of patients with dementia: design of a randomized controlled trial. BMC Geriatr 8: 2. 18208607 10.1186/1471-2318-8-2 PMC2259355

[17] Sheehan DV, Lecrubier Y, Sheehan KH, Amorim P, Janavs J, et al (1998) The Mini-International Neuropsychiatric Interview (M.I.N.I.): the development and validation of a structured diagnostic psychiatric interview for DSM-IV and ICD-10. J Clin Psychiatry 59 Suppl 2022–33. 9881538

